# Evaluating the Sapien® XT Valve in Native Right Ventricular Outflow Tracts After Tetralogy of Fallot Repair: Mid- and Long-Term Results

**DOI:** 10.1007/s00246-025-03776-x

**Published:** 2025-01-21

**Authors:** Ender Odemis, Aydin Celikyurt, Mete Han Kizilkaya, İbrahim Halil Demir

**Affiliations:** 1 Department of Pediatric Cardiology, Faculty of Medicine, Koc University, Istanbul, Turkey; 2 Department of Pediatrics, Faculty of Medicine, Erciyes University, Kayseri, Turkey; 3 Dr. Siyami Ersek Thoracic and Cardiovascular Surgery Training and Research Hospital, Istanbul, Turkey

**Keywords:** Percutaneous pulmonary valve implantation, Sapien® XT valve, Right ventricular outflow tract, Fallot tetralogy, Pulmonary regurgitation

## Abstract

Although the long-term outcomes of the surgical grafts are well defined and reported, the data regarding the mid-and long-term results of the balloon-expandable percutaneous valves in the native right ventricular outflow tract (RVOT) is limited. We retrospectively evaluated 42 patients who underwent PPVI (Sapien® XT valve) to native RVOT due to severe pulmonary regurgitation (PR) and/or moderate to severe pulmonary stenosis (PS) between August 2015 and November 2020. The median patient age at the time of PPVI was 13.4 years (6.1–36.5 years). The median body weight of the patients was 42 kg (15–110 kg). The rate of patients who were followed up without the need for percutaneous or surgical intervention was 97.4% at the end of year 1, 89.3% at the end of year 3, and 85.8% at the end of year 5. At the end of year 6, the proportion of patients requiring no procedure remained constant, with year 5 at 85.8%, but decreased to 70.2% at the end of year 7. Although the early results are very encouraging, it is seen that PPVI in patients with RVOT in the long term brings some problems. The most important of these is tricuspid valve problems, which were not considered before the procedure. Patients requiring reintervention due to pulmonary regurgitation show similar characteristics to surgical valves’ long-term results.

## Introduction

Tetralogy of Fallot (TOF) is the most common cyanotic congenital heart disease (CHD). With advancements in surgical techniques and intensive care services, the mortality rate of TOF has decreased to less than 1% in many centers. Consequently, the number of adult patients who have undergone total repair of TOF has steadily increased worldwide. Long-term follow-up of these patients, particularly those reaching the second decade after total repair, has revealed several late complications associated with surgical intervention, including right heart failure, severe arrhythmias, and sudden death [1–3]. Severe pulmonary regurgitation (PR), largely attributed to the transannular patch used during surgical repair, has been identified as a primary factor underlying these complications. Initially considered a benign lesion, PR is now recognized as a condition requiring treatment [3, 4]. To address PR, various surgical approaches using conduits, such as xenografts, homografts, and bioprostheses, have been employed for pulmonary valve replacement. Percutaneous pulmonary valve implantation (PPVI) was developed as a minimally invasive alternative to traditional surgical approaches for managing conduit dysfunctions. Bonhoeffer et al. [5] reported the first percutaneous implantation in a surgically implanted xenograft, which marked the beginning of PPVI therapy. This innovation led to the development of the Melody® valve (Medtronic), constructed by incorporating a bovine jugular vein into a Cheatham platinum® stent specifically designed for implantation in conduits. The Melody® valve received the European CE mark in 2006 and its first implantation in the USA occurred in January 2007. In 2010, the valve received FDA approval for implantation in conduits through the humanitarian device exemption guidelines and gained full premarket approval in January 2015 [6]. However, due to its smaller size, the Melody® valve was limited to use in a small subset of patients with native, enlarged right ventricular outflow tract (RVOT) following TOF repair [7]. The Edwards Sapien® transcatheter heart valve (Edwards Lifesciences, Irvine, California), initially designed for aortic valve implantation, has since been adapted for use in conduits and native, enlarged RVOT [8, 9]. The second-generation Sapien® valves, including the XT® and Sapien 3® (S3), consist of bovine pericardial leaflets mounted on a cobalt–chromium, stainless steel, and balloon-expandable frame. These valves are available in larger diameters (26 mm and 29 mm) compared to the Melody® valve, enabling their application in patients with large native or patched RVOT. The Sapien® XT valve has been used in the pulmonary position since 2006 and received CE certification in 2010. Several studies have documented its feasibility and promising early outcomes in native RVOT [10–13]. While the long-term outcomes of surgical grafts are well documented, data on the durability and effectiveness of balloon-expandable percutaneous valves, such as the Sapien® XT, in native RVOT remain limited. This study aims to present the mid- and long-term results of the Sapien® XT valve in patients with native RVOT following repaired TOF with transannular patch.

## Method

In our study, we retrospectively evaluated 42 patients who underwent PPVI for native RVOT due to severe pulmonary regurgitation (PR) and/or moderate to severe pulmonary stenosis (PS) between August 2015 and November 2020 at Koc University Hospital in Istanbul, Turkey. Demographic characteristics, details of the PPVI procedure, and pre- and post-procedural data, including medical history, physical examination findings, New York Heart Association (NYHA) classification, electrocardiography (ECG), and echocardiography (ECHO) results, as well as 24-h rhythm Holter monitoring and cardiac magnetic resonance imaging (MRI) findings (when available), were analyzed. The ethics committee approval for this study was obtained from the Koc University Ethics Committee (approval number: 2022.434.IRB1.160).

### Patient Selection

Patients with prior surgical conduits or bioprostheses were excluded from the study. Only those who had undergone TOF repair using a transannular patch and retained a native RVOT were included. The indications for valve implantation were determined based on the College of Cardiology/American Heart Association (ACC/AHA) guidelines [14]. The primary indication was the presence of unexplained cardiovascular symptoms in patients with severe pulmonary regurgitation following complete corrective surgery. In asymptomatic patients, the pulmonary regurgitation (PR) fraction was assessed using cardiac MRI. PR fraction was categorized as mild (< 20%), moderate (20–40%), or severe (> 40%) [15]. In asymptomatic patients with moderate to severe pulmonary regurgitation, PPVI was performed if two or more of the following criteria were met:

1. Mild to severe right or left ventricular dysfunction in MRI,
2. Severe right ventricular dilatation observed on MRI (RVESVi ≥ 80 mL/m^2^ and/or RVEDVi ≥ 160 mL/m^2^),
3. Right ventricular end-diastolic volume at least twice that of the left ventricular end-diastolic volume on MRI,
4. Right ventricular systolic pressure equal to or exceeding two-thirds of systemic pressure,
5. QRS duration ≥ 140 ms on electrocardiogram [16],
6. An objectively documented progressive decline in exercise capacity.

### Demographic Characteristics

Patient characteristics, including gender, underlying cardiac conditions, age and body weight at the time of PPVI, pre-procedural clinical findings, and New York Heart Association (NYHA) classification, were analyzed.

### Material and Pre-procedure Evaluation

In our study, the Sapien XT® balloon-expandable valve system (Edwards Lifesciences Inc.) was utilized. The Sapien XT® valve, available in four diameters (20 mm, 23 mm, 26 mm, and 29 mm), has been used in the pulmonary position since 2006 and obtained CE certification in 2010. The Sapien XT® valve features a radiopaque, stainless steel, balloon-expandable support structure integrated with a unidirectional, triple-leaflet bovine pericardium valve, and a polyethylene terephthalate fabric cuff. The Novoflex® delivery system was utilized for implantation.

Each patient scheduled for PPVI underwent pre-procedural evaluation with electrocardiography (ECG) and echocardiography. Cardiac MRI was performed in all clinically asymptomatic patients, while both cardiac CT and MRI were conducted in symptomatic patients as clinically indicated. The right ventricular outflow tract, pulmonary annulus, main pulmonary artery, bifurcation, and branch pulmonary artery diameters were thoroughly assessed using echocardiography. In addition, the severity of pulmonary valve stenosis and regurgitation, tricuspid valve regurgitation, and aortic valve regurgitation, along with the estimated right ventricular systolic pressure and overall cardiac function, were documented.

The severity of pulmonary regurgitation (PR) was assessed through echocardiography using specific echocardiographic criteria. Color Doppler imaging was used to identify a broad regurgitant jet occupying a substantial portion of the pulmonary artery, with a regurgitation fraction generally exceeding 50%. Continuous wave Doppler was utilized to assess elevated diastolic flow velocities in the pulmonary artery extending across the pulmonary valve. Additionally, pronounced retrograde flow from the pulmonary artery into the right ventricle during diastole was considered a strong indicator of severe PR. These comprehensive criteria were retrospectively applied to ensure accurate classification of severe PR and to guide the evaluation of treatment outcomes [17].

These data were integral in determining the appropriate pulmonary valve size for implantation. Patients with a RVOT diameter of 27 mm or larger at its narrowest point, as assessed by echocardiography, were deemed unsuitable for PPVI. Such patients were referred for surgical valve replacement to ensure optimal outcomes.

### Catheterization and Percutaneous Pulmonary Valve Implantation

**Fig. 1:**
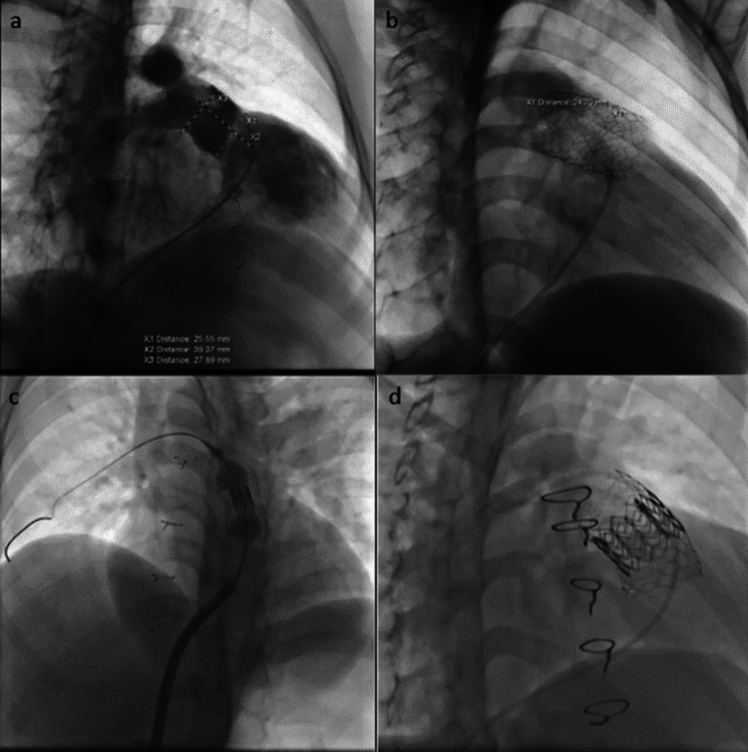
Stages of the PPVI procedure to the native RVOT. **a** Identification of pulmonary arteries and valve regurgitation on RVOT angiography. **b** Stent implantation (prestenting). **c** Valve implantation. **d** Final imaging of the implanted valve within the stent

All procedures were performed under deep sedation without intubation. Prophylactic cefazolin (50 mg/kg) was administered one hour prior to the procedure. After vascular access was established via femoral arteries and veins, anticoagulation was achieved with heparin to maintain an activated clotting time (ACT) between 150 and 250 s. The length and narrowest diameter of the main pulmonary artery were measured using angiograms in the cranial right anterior oblique and left lateral positions. An Amplatzer® sizing balloon (AGA) test was conducted to assess coronary artery compression and a simultaneous right ventricular (RV) angiogram was performed to evaluate contrast passage through the RVOT. Following these assessments, details such as the time interval between stent and valve implantation, the type and diameter of the valve, and the length of the stent were recorded (Fig. 1).

Two procedures were performed on patients with enlarged RVOT without evidence of stenosis in the RVOT or main pulmonary artery. If a narrowed segment or indentation was detected during balloon sizing or following stent implantation, the valve was implanted in the same session.

Additional procedures, including stent pre-dilatation, peripheral pulmonary angioplasty, and the use of additional stents, were documented. After PPVI, patients underwent immediate evaluation using echocardiography in the catheterization room. Key parameters such as valve position, the severity of stenosis and regurgitation, tricuspid valve regurgitation, and any potential complications were assessed and recorded.

Clinically successful PPVI was defined as a procedure resulting in mild or less pulmonary regurgitation and a peak pulmonary stenosis gradient of < 15 mmHg on discharge echocardiography.

### Follow-up

Following the PPVI procedure, patients were evaluated on postoperative 1 day, 1 month, 3 months, 6 months, 1 year, and annually thereafter. The 1st year after the procedure was classified as a short-term follow-up, 1 to 5 years as the mid-term follow-up, and ≥ 5 years as a long-term follow-up. Due to the limited availability of long-term follow-up data in the literature, the 6th- and 7th-year post-procedure were analyzed separately. Routine evaluations included electrocardiography (ECG) and echocardiography, while 24-h rhythm Holter monitoring was performed for patients reporting symptoms, such as palpitation or syncope. Starting from the 2nd year of follow-up, cardiac MRI was scheduled for eligible patients to enable a more detailed evaluation of cardiac function.

### Echocardiography and Cardiac Magnetic Resonance Imaging

During the follow-up of patients after PPVI, echocardiographic imaging was performed at every visit, and detailed assessments were made of the RVOT, pulmonary annulus, main pulmonary artery, bifurcation, and pulmonary artery branches. Additionally, the severity of pulmonary valve stenosis and regurgitation, tricuspid valve regurgitation, estimated right ventricular systolic pressure (derived from tricuspid regurgitation), aortic valve regurgitation, and overall cardiac function were documented. The primary hemodynamic outcome measures assessed via echocardiography included the degree of pulmonary regurgitation, peak pulmonary stenosis gradient, and the severity of tricuspid regurgitation. The degree of pulmonary regurgitation was categorized into four groups: none/trace, mild, moderate, and severe. The peak pulmonary stenosis (PS) gradient was classified as follows: none, mild (16–35 mmHg), moderate (36–63 mmHg), and severe (≥ 64 mmHg) [18]. The severity of tricuspid regurgitation was categorized as none, mild, moderate, or severe [19].

Cardiac MRI was performed either prior to valve implantation or during post-procedural follow-up, when clinically necessary and the patient was eligible. RVEDV index, RVESV index, right ventricular ejection fraction, PR fraction, and right ventricle/left ventricle (RV/LV) volume ratio were analyzed.

### Statistical Analysis

The statistical analyses were conducted using SPSS 28.0 (Chicago, IL, USA). Continuous variables were summarized as medians with minimum and maximum values, while categorical variables were presented as counts and percentages. The Shapiro–Wilk test was used to assess the normality of the data, and nonparametric tests were applied for variables that did not follow a normal distribution. The Friedman test was employed to evaluate changes within dependent groups over time. For statistically significant differences, pairwise comparisons were performed using the Wilcoxon signed rank test. A *p* value less than 0.05 was considered statistically significant. Survival curves and analyses were generated using the Kaplan–Meier method.

## Results

The study included 42 patients who underwent percutaneous pulmonary valve implantation (PPVI) between August 2015 and November 2020. Among the patients, 26 were male and 16 were female. The median age at the time of PPVI was 13.4 years (6.1–36.5 years), and the median body weight was 42 kg (15–110 kg). All participants in the study had a diagnosis of tetralogy of Fallot (TOF) and had previously undergone surgical repair with a transannular patch.

### Pre-procedural Imaging

All patients underwent pre-procedural evaluation with echocardiography, and cardiac MRI was also performed in all asymptomatic patients, as well as in symptomatic patients who were eligible for MRI. Pulmonary regurgitation was classified as moderate to severe, with a PR fraction of 40%, in two patients, while all other patients had severe PR. Among these, 10 patients (23.8%) exhibited a mixed lesion, characterized by the presence of pulmonary regurgitation accompanied by pulmonary stenosis. The median RVOT diameter prior to valve replacement was 21 mm (15–27 mm).

Before the procedure, 35 of the 42 patients were symptomatic, whereas 7 patients remained asymptomatic. Cardiac MRI was performed in all asymptomatic patients and in 6 of the symptomatic patients. Pre-procedural cardiac MRI measurements revealed a median right ventricular end-diastolic volume (RVEDV) index of 136 mL/m^2^ (109–170 mL/m^2^), a median right ventricular end-systolic volume (RVESV) index of 82 mL/m^2^ (68–126 mL/m^2^), a median right ventricular ejection fraction (EF) of 40% (22%–80%), a median PR fraction of 49% (40%–55%), and a median RV/LV volume ratio of 1.9 (1.4–2.7) (Table 1). Therefore, patients who did not meet the procedural indication based on a single parameter underwent PPVI because they met at least two other parameters.

**Table 1:** Demographic and pre-procedural imaging data of the TOF patients with transannular patch

Demographic data	Patient count (*n*/%) or median (minimum–maximum)
Age (years)	13.4 (6.1–36.5)
Gender	
Male	26 (61.9)
Female	16 (38.1)
Body weight (kg)	42 (15–110)
Clinical condition	
Symptomatic	35 (83.3)
Asymptomatic	7 (26.7)

Echocardiography findings	Patient count (*n*/%) or median (minimum–maximum) (*n* = 42)
RVOT dysfunction type	
Pulmonary regurgitation	32 (76.2)
Pulmonary regurgitation + pulmonary stenosis	10 (23.8)
RVOT diameter (mm)	21 (15–27)
Pulmonary regurgitation	
None, trace	0 (0)
Mild	0 (0)
Moderate	2 (4.8)
Severe	40 (95.2)
Pulmonary stenosis	
None, mild	32 (76.1)
Moderate	8 (19)
Severe	2 (4.8)
Tricuspid regurgitation	
None, mild	37 (88.1)
Moderate	5 (11.9)
Severe	0 (0)

Cardiac MRI findings	Median (minimum–maximum) (*n* = 13)
RVEDVi (ml/m^2^)	136 (109–170)
RVESVi (ml/m^2^)	82 (68–126)
RV EF (%)	40 (22–80)
PR fraction (%)	49 (40–55)
RV/LV volume	1.9 (1.4–2.7)

### Stent and Valve Implantation

Stent and pulmonary valve implantation were performed in two separate sessions with prestenting in 34 out of 42 patients (80.9%), in a single session in 7 patients (16.7%), and using the hybrid method in 1 patient (2.4%). Among the 34 patients who underwent stent and valve implantation in two sessions, the median interval between the procedures was 90 days (34–270 days).

A total of 47 stents were successfully implanted in 42 patients. AndraStent® (Andramed, Reutlingen, Germany) was used in all procedures. The stent was successfully implanted on the first attempt in 40 out of 42 patients (95.2%). In 2 patients, the stent implantation was unsuccessful on the first attempt due to uncertainty regarding the narrowest diameter of the RVOT. These patients were subsequently re-evaluated using echocardiography and cardiac CT, after which prestenting was successfully performed. The median stent length was 43 mm (22–57 mm).

Successful PPVI was achieved on the first attempt in 40 out of 42 patients (95.2%). One patient was referred for surgical intervention and underwent bioprosthetic valve implantation after two unsuccessful attempts at stabilizing the wire for pulmonary valve implantation. In another patient, despite attempts to access the pulmonary artery through both the femoral and jugular veins, access could not be achieved. Following these unsuccessful attempts, the family was informed in detail about the situation, including the procedural challenges and alternative options. With their informed consent, the decision was made to proceed with the hybrid method. This approach enabled successful implantation of the valve, providing a viable solution to the anatomic and procedural challenges encountered. After a second attempt, PPVI was successfully completed in 41 out of 42 patients (97.6%). The median diameter of the implanted pulmonary valves was 26 mm (23–29 mm) (Table 2).

**Table 2:** Procedure-related features

Procedural characteristics	Patient count (*n*/%) or median (minimum–maximum)
Procedure type	
Two sessions	34 (80.9)
Single session	7 (16.7)
Hybrid	1 (2.4)
Number of implanted stents (AndraStent®)	
1	37 (88.1)
2	5 (11.9)
Stent length (mm)	43 (26–57)
Time between stent and valve implantation (day)	90 (34–270)
Valve diameter	
23 mm	14 (33.3)
26 mm	21 (50)
29 mm	7 (16.7)

### Early Complications After PPVI

Early complications were observed in 2 patients (4.7%). In one patient, pulsed ventricular tachycardia was observed during the procedure; cardioversion was performed first, followed by the administration of amiodarone infusion. Postprocedurally, amiodarone infusion was continued for 3 days, followed by a gradual tapering and discontinuation of the medication. No arrhythmias were detected thereafter. In the second patient, inotropic therapy was initiated due to hypotension during the procedure. The patient was monitored in the intensive care unit, where blood pressure stabilized and returned to normal within 1 day. Stent dislocation, stent fracture, or periprocedural mortality were not observed.

### Follow-up

**Fig. 2:**
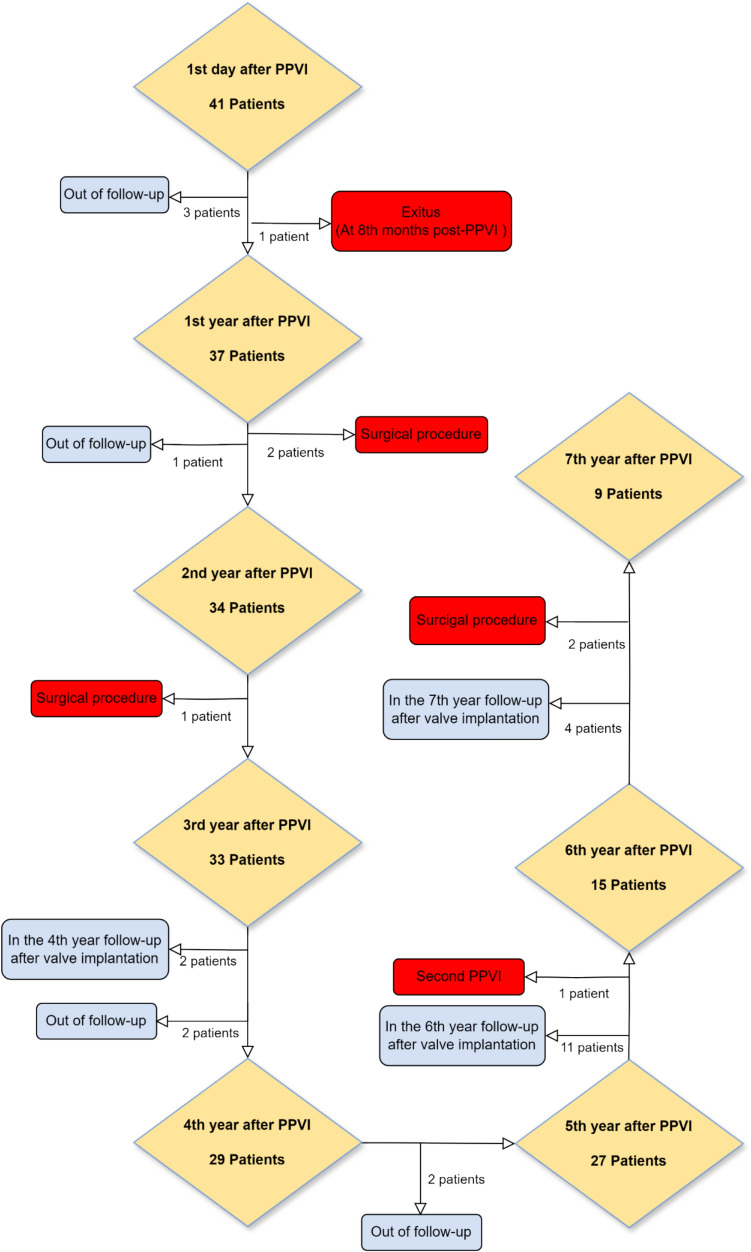
Patient follow-up timeline after PPVI

Excluding one failed procedure, a total of 41 patients who underwent PPVI were followed up. The follow-up rates were as follows: 41 patients (100%) on the 1st day, 37 patients (90.2%) at 1 year, 34 patients (82.9%) at 2 years, 33 patients (80.4%) at 3 years, 29 patients (70.7%) at 4 years, 27 patients (65.8%) at 5 years, 15 patients (36.5%) at 6 years, and 9 patients (21.9%) at 7 years. During the follow-up period, 3 patients were excluded from the study after the 1st day, 1 patient after the 1st year, 2 patients after the 3rd year, and 2 patients after the 4th year due to irregular attendance at outpatient clinic visits. Additionally, patients were regularly contacted by phone throughout the follow-up period to obtain updates on their clinical status (Fig. 2).

After excluding one patient who underwent failed PPVI and was referred for surgery, as well as three patients who were lost to follow-up after discharge on the 1st-day post-PPVI, the median follow-up duration was 60 months (8–84 months).

### Post-procedural Valve Functions

The degree of pulmonary regurgitation, peak gradient of pulmonary stenosis, and tricuspid regurgitation were monitored in the patients’ postoperative 1st day and subsequent annual echocardiograms (Table 3).

**Table 3:** Follow-up of PR grade, PS peak gradient, and TR grade after PPVI

ECHO findings	1st day after PPVI	1st year after PPVI	3rd year after PPVI	5th year after PPVI
Pulmonary regurgitation				
None/trace	38 (92.7%)	26 (70.3%)	16 (48.5%)	6 (22.2%)
Mild	3 (7.3%)	10 (27%)	15 (45.4%)	17 (63%)
Moderate	0 (0%)	1 (2.7%)	2 (6.1%)	4 (14.8%)
Severe	0 (0%)	0 (0%)	0 (0%)	0 (0%)
Pulmonary stenosis peak gradient (mm Hg)	8 (5–15)	12 (5–22)	16 (5–26)	23 (8–34)
Tricuspid regurgitation				
None, mild	39 (95.2%)	35 (94.6%)	32 (96.9%)	26 (96.3%)
Moderate	2 (4.8%)	2 (5.4%)	1 (3.1%)	1 (3.7%)
Severe	0 (0%)	0 (0%)	0 (0%)	0 (0%)

The degree of pulmonary regurgitation (PR) measured by echocardiography on the 1st-day, at 1-year, 3-year, and 5-year post-procedure was analyzed using the Friedman test, which revealed a statistically significant difference among the groups (*p* < 0.001). Subsequent pairwise comparisons, conducted using the Wilcoxon test, demonstrated a statistically significant increase in PR severity between the 1st day and 1 year, 1 year and 3 years, and 3 years and 5 years (*p* = 0.002, *p* = 0.002, and *p* = 0.006, respectively).

The peak gradient of pulmonary stenosis (PS) measured by echocardiography on the 1st-day, at 1-year, 3-year, and 5-year post-procedure was analyzed using the Friedman test, revealing a significant difference among the groups (*p* < 0.001). Pairwise comparisons, performed using the Wilcoxon test, indicated a statistically significant increase in the PS peak gradient between the 1st day and 1 year, 1 year and 3 years, and 3 years and 5 years (*p* < 0.001, *p* = 0.004, and *p* < 0.001, respectively).

Moderate tricuspid regurgitation (TR) was identified in 5 patients prior to PPVI, which was attributed to right ventricular volume overload caused by severe PR. Following PPVI, moderate TR resolved in all these patients. However, 2 patients developed new moderate TR due to tricuspid valve damage associated with the procedure. When the degree of TR measured by echocardiography on the 1st-day, at 1-year, 3-year, and 5-year post-procedure was analyzed using the Friedman test, no statistically significant difference was observed among the groups (*p* = 0.212).

### Cardiac Magnetic Resonance Findings after PPVI

Cardiac MRI was performed on ten patients who underwent PPVI, both before the procedure and at long-term follow-up. Comparison of pre- and post-PPVI parameters using the Wilcoxon test revealed statistically significant improvements in the RVEDV index (*p* = 0.047), RVESV index (*p* = 0.047), PR fraction (*p* = 0.005), and RV/LV volume ratio (*p* = 0.009). However, no statistically significant difference was observed in the right ventricular EF (*p* = 0.286) (Table 4).

**Table 4:** Data of patients who underwent cardiac MRI before and after PPVI

Cardiac MRI parameters	Before procedure	After procedure	*p* value
RVEDVi (ml/m^2^)	136 (109–170)	120.5 (81–157)	0.047
RVESVi (ml/m^2^)	80.5 (70–126)	72 (44–84)	0.047
RV EF (%)	40 (22–80)	41 (38–57)	0.286
PR fraction (%)	49 (40–55)	4.1 (0–29)	0.005
RV/LV volume	1.9 (1.4–2.7)	1.4 (1–1.9)	0.009

### Late Complications After PPVI

Two patients with mild tricuspid regurgitation before PPVI developed moderate TR from day 1 post-procedure (4.8%). The newly developed TR in these two patients was found to be due to tricuspid valve damage during the procedure. Both patients developed moderate pulmonary regurgitation during follow-up (one in the 2nd year and the other in the 7th year) and became symptomatic without any other identifiable cause, necessitating surgical pulmonary valve replacement and tricuspid repair.

Arrhythmia developed in three patients (7.3%) during the mid-to-long-term follow-up. One patient complained of palpitations in the 2nd year after the procedure and a 24-h rhythm Holter was performed. Ventricular extrasystole was observed, and medical treatment was started. The treatment was reduced and discontinued in the follow-up of the patient whose complaint regressed with medical treatment. One patient experienced palpitations, fatigue, and syncope attacks in the 4th year after the procedure. A 24-h rhythm Holter test showed ventricular extrasystoles and intermittent pauses lasting longer than 2 s and an ICD was implanted. In the last patient, a pacemaker was implanted in the 6th year of follow-up due to a complete AV block that developed after a COVID-19 infection.

Our study observed no infective endocarditis (IE) or stent fracture during follow-up. The total complication rate during follow-up was 12.1%.

### Reinterventions

During the 2nd-year follow-up, 2 patients underwent surgery. One of the patients had no pulmonary regurgitation on post-procedural day 1, but moderate PR was detected at the 1-year follow-up. A second PPVI was recommended for this patient who was symptomatic and whose clinical complaints could not be explained by any other cause, but the patient was referred to surgery because the family did not accept the second PPVI. The other patient, who had mild tricuspid regurgitation before the procedure, developed moderate TR by the 1st-day post-procedure. At the 1st-year follow-up, mild PR was added to moderate TR, and by the 15th month, severe TR and moderate PR were observed. Additionally, the patient became clinically symptomatic and subsequently underwent pulmonary valve replacement and tricuspid repair.

One patient who underwent surgery in the 3rd year of follow-up, newly developed moderate pulmonary regurgitation was observed during the 2nd year of follow-up. Although the patient was asymptomatic, he presented with the following cardiac MRI findings: RVEDVi: 171 mL/m^2^, RVESVi: 95 mL/m^2^, RVEF: 44%, and an RV/LV volume ratio: 2.3. Based on these findings, surgical valve replacement was performed.

One of the two patients who underwent surgery in the 7th year of follow-up showed mild PR in the 4th year and moderate PR in the 6th year after PPVI. The patient became symptomatic with exertional dyspnea and subsequently underwent surgery. The other patient, who did not have tricuspid regurgitation before PPVI but developed moderate tricuspid regurgitation on the 1st day after the procedure, became symptomatic and underwent surgical valve replacement and tricuspid repair after moderate pulmonary regurgitation was observed in the 6th year of follow-up.

In the patient who underwent a second PPVI during the 6th year of follow-up, moderate PR was detected starting from the 3rd-year post-procedure. The patient remained asymptomatic until the 5th-year post-procedure but developed symptoms, thereafter, leading to the decision for a second PPVI (Table 5).

**Table 5:** Summary of reinterventions, including reasons, timing, and types of procedures performed during follow-up

Patient	Reason for intervention	Intervention time	Intervention type
Patient 1	Moderate PR and symptoms that could not be attributed to any other cause	13th month	Surgical valve replacement
Patient 2	Severe tricuspid regurgitation and moderate pulmonary regurgitation with symptoms that could not be attributed to any other cause	15th month	Surgical valve replacement and tricuspid repair
Patient 3	Moderate pulmonary regurgitation with RVEDVi: 171 ml/m^2^, RVESVi: 95 ml/m^2^, RVEF: 44%, RV/LV ratio: 2.3	30th month	Surgical valve replacement
Patient 4	Symptomatic progression of moderate PR	69th month	Second PPVI
Patient 5	Exertional dyspnea with progression from mild PR to moderate PR	74th month	Surgical valve replacement
Patient 6	Moderate PR with moderate TR and the patient became symptomatic	78th month	Surgical valve replacement and tricuspid repair

### Survival Without Intervention

**Fig. 3:**
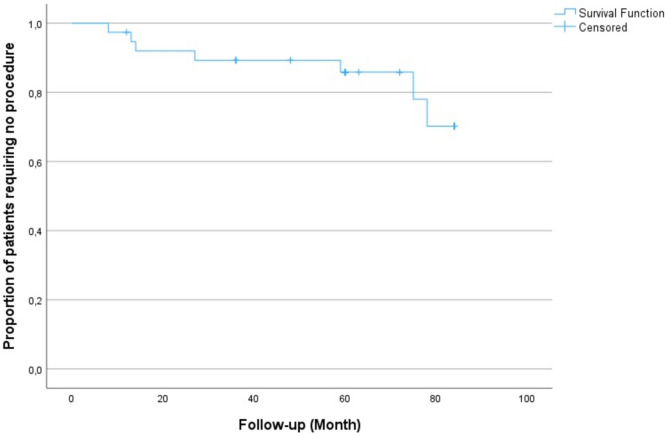
Proportion of patients not requiring percutaneous or surgical procedures after PPVI (Kaplan–Meier curve)

The time curve of patients after valve implantation without the need for percutaneous or surgical procedures was plotted using the Kaplan–Meier method. Mortality was observed in one patient at the 8th month of follow-up after PPVI; however, this was unrelated to the procedure or valve complications. No additional mortality occurred during the study period. The proportion of patients who did not require any further interventions was 97.4% at the end of the 1st year, 89.3% at the end of the 3rd year, and 85.8% at the end of the 5th year. By the 6th year, this proportion remained steady at 85.8%, but it declined to 70.2% by the end of the 7th year. The time to reach the 75th percentile for patients without requiring additional procedures was calculated as 6.5 years (Fig. 3).

## Discussion

In the modern era, surgical repair of TOF has a minor rate of mortality and early morbidity. Concerning this, the number of patients who reach adulthood constantly increases [1]. However, late complications and sudden death are coming to the eyesight that is caused by severe PR due to transannular patches. Traditionally, surgical bioprosthesis implantation has been applied to solve PR. Repeated surgeries lead to complications of surgery and even increased mortality [20]. Percutaneous pulmonary valve implantation emerged as an alternative way of the treatment of severe PR and is used more commonly. It has been used in patients with surgically implanted conduits and bioprosthesis. Using the PPVI for patients with transannular patches in the native RVOT is a relatively new way of treatment. Several reports show early feasibility and the results of the PPVI in the native RVOT [7, 11, 21]. In the presented study, early results of the PPVI, such as implantation success and low complication rate, were also satisfying compared to the literature. A significant PR was not observed in any of the patients immediately after implantation, and in all patients, PR was eliminated successfully. Right ventricle pressure was also reduced in all patients after PPVI due to relieving pulmonary arterial stenosis. According to the data reported above, we can consider that early feasibility, efficiency, and safety are promising for the PPVI with Edwards XT ® valve in native RVOTs.

On the other hand, balloon-expandable valves were initially designed for the aortic position and adapted to the pulmonic position. Therefore, their performance is still questioned in the pulmonic position, a quietly different hemodynamic condition from the aorta. This study is essential for giving insight into the mid- and long-term performance of balloon-expandable valves in native RVOTs and transannular patches.

### Pulmonary Regurgitation

We detected a statistically significant increase in the PR within time after implantation. At the end of the 3rd year, three patients (7.3%) required surgical valve replacement and/or moderate PR with concomitant TR. In the remaining patients, although the PR appeared to reach 51.9%, none was at the severity of significance, which does not require reintervention or replacement. A prominent increase in mild PR occurred in the 1st year of the implantation. This means the patients who need interventions in future are becoming evident in the 1st year of implantation. Therefore, we recommend closely following those patients who had PR in the early stage for the possibility of reintervention, even if it is mild initially.

### Pulmonary Stenosis

Pulmonary stenosis is an influencing factor that affects the long-term outcome of valve competency in the PPVI procedure [22]. In this study, PS was not the main problem, and some patients had PS in addition to PR. Therefore, decreasing RV pressure and gradient was not the main issue. Consequently, we achieved a reasonable decrease in the RV pressure in all patients. However, we observed a gradual increase in the RV pressure due to the calcification of the valve leaflets. On the other hand, none of our patients had to undergo surgery or reintervention due to pure PS. Therefore, we believe that PS is not the leading cause of those patients with severe PR after TOF repair with the transannular patch, especially in cases of severe RV pressure does not present before the procedure.

### Tricuspid Regurgitation

Tricuspid regurgitation is a recognized complication that may occur during the procedure. Despite careful attention by physicians to minimize the risk of tricuspid valve leaflet damage through techniques, such as cautious balloon catheter maneuvers, the rigid delivery system of the Edwards XT® valve can still result in tricuspid valve injury, leading to moderate to severe TR. In our series, two patients required surgical repair of TR along with concomitant pulmonary valve replacement. We believe that TR represents a significant yet underrecognized issue associated with the procedure, with a notable impact on long-term reintervention and surgical requirements for affected patients. Following the initial experience with PPVI, advancements were made to address the challenges posed by the Retroflex® and Novoflex® delivery systems, leading to the development of improved delivery systems in subsequent valve generations. Additionally, the use of Gore DrySeal® long sheaths has been strongly recommended by some physicians as a strategy to mitigate the risk of TR damage during the procedure [23].

### MRI Findings and RV Functions

Since most of our patients had symptoms at admission, we did not obtain an MRI examination for all patients. Therefore, the data in this study is not satisfying in showing the influence of the PPVI on the RV functions of the PPVI in patients with native RVOT. However, this limited data aligns with the literature, showing a reduction in right ventricular end-systolic and end-diastolic volumes, but no significant improvement in right ventricular EF. This may be attributed to the late admission of our patients, with symptoms becoming apparent only after right ventricular (RV) function had deteriorated to an irreversible state. MRI studies also show that strain findings of the patient after PPVI do not change, and this may interpret as delayed procedure timing for the PPVI [24, 25]. Nevertheless, we believe that the results of this study provide valuable insights into reconsidering the timing of PPVI, suggesting that earlier intervention may lead to better improvements in right ventricular function.

### Arrhythmia

In the study, no rhythm disturbances were observed during the procedure, except for one patient who developed ventricular tachycardia (VT) and received appropriate treatment. During mid- and long-term follow-up, medical therapy was required in one patient due to ventricular arrhythmias, an implantable cardioverter-defibrillator (ICD) was implanted in another patient, and a pacemaker was implanted in a third patient due to complete atrioventricular (AV) block following a COVID-19 infection. Recent studies have highlighted the prevalence and management of ventricular arrhythmias in patients with repaired Tetralogy of Fallot (TOF). Approximately, 20–50% of patients with TOF experience tachyarrhythmias and bradyarrhythmia’s, often necessitating the use of cardiac implantable electronic devices (CIEDs), such as pacemakers and implantable cardioverter defibrillators [26]. Additionally, patients with a history of ventricular tachycardia or left ventricular dysfunction undergoing pulmonary valve replacement are at a higher risk for arrhythmic events post-operation [27].

Especially at the beginning of the experiences, we preferred to use longer stents in the RVOT to avoid dislocations. This stent may come in contact with the RVOT muscle bundles to cause seldom ventricular extrasystoles or arrhythmias. In this study, the Sapien XT ® valve has been reported since no approved Edwards S3 ® next-generation valve was available. After the S3 ® valve came into the market due to its longer structure, which is safer for implantation without stenting, some studies have been reported regarding the feasibility of the PPVI without presenting [12].

### Infective Endocarditis

We did not see any patients with infective endocarditis. This complies with the literature that reported a low rate of IE with the Edwards Sapien and XT and valves [28, 29]. We consider all patients before the procedure because of dental problems, which may be closely related to early infective endocarditis. We also recommend that all patients have dental examinations yearly to avoid IE complications.

### Hybrid Method in PPVI

In patients where the transcatheter approach is not feasible and surgical valve replacement poses significant risks, the hybrid method emerges as a viable alternative. In our series, one patient could not undergo the procedure via the transcatheter route, and valve implantation was successfully achieved using the hybrid method. The indications for the hybrid method include the inability to achieve suitable vascular access for the transcatheter procedure, technical issues related to sheaths, guidewires, and catheters necessary for the transcatheter approach, a complex right ventricular outflow tract anatomy with multiple associated abnormalities, or the absence of an adequate landing zone for valve placement. Ultimately, the hybrid approach stands as a viable option that should be considered when conventional methods are insufficient or pose significant challenges [30–32].

### Limitations

This retrospective study examines the mid- and long-term results of using balloon-expandable valves in native RVOT. Because of the late presentation of many patients, RV volume and function were severely impaired, and patients were symptomatic. Therefore, the study cannot provide sufficient information regarding MRI findings. In addition, some of the complications mentioned in this article are much less common with newer systems due to the increase in experience and improvements in the valve and delivery system in the 9 years since the first case was performed.

In conclusion, although the early results are very encouraging, it is seen that PPVI in patients with RVOT in the long term brings some problems. The most important of these is tricuspid valve problems, which were not considered before the procedure. With improvements in valves and delivery systems and increasing experience, it will be possible to prevent these complications. Patients requiring reintervention due to pulmonary regurgitation show similar characteristics to surgical valves’ long-term results. Prospective extensive series cohort studies are needed to understand the long-term effects of PPVI in RVOT on right ventricular function.

## Data Availability

No datasets were generated or analysed during the current study.
